# Hepatitis B Infection and Mother-to-Child Transmission in Haiphong, Vietnam: A Cohort Study with Implications for Interventions

**DOI:** 10.1155/2020/4747965

**Published:** 2020-08-20

**Authors:** Pham Minh Khue, Nguyen Thi Thuy Linh, Vu Hai Vinh, Luu Vu Dung, Bang Nguyen Van

**Affiliations:** ^1^Faculty of Public Health, Haiphong University of Medicine and Pharmacy, Haiphong, Vietnam; ^2^Department of Tropical and Infectious Diseases, Viet-Tiep Hospital, Haiphong, Vietnam; ^3^Department of Biochemistry, Haiphong Gyneco-Obstetric Hospital, Haiphong, Vietnam; ^4^Department of Pediatrics, Hanoi Medical University, Hanoi, Vietnam

## Abstract

**Background:**

There is little data available on HBV infection and mother-to-child transmission (MTCT) in Vietnam.

**Objective:**

This study is aimed at assessing the prevalence of HBV infection and the current situation of MTCT in Haiphong, Vietnam.

**Methods:**

A transversal survey of 1721 pregnant women followed by an observational prospective cohort study of 183 HBV-infected women was conducted at Haiphong Gyneco-Obstetric Hospital. Women were followed up up to 12-month postpartum; use of prevention measures and the MTCT rate were evaluated. HBV infection in children was defined by a HBsAg-positive test at 12 months of age.

**Results:**

At baseline, 183 of 1721 pregnant women (10.6%) tested HBsAg positive. Among them, 23.0% were HBeAg positive, 26.2% had a detectable load of HBV DNA, and 13.1% had a HBV DNA load ≥ 200,000 IU/mL. All women underwent MTCT prevention antiviral therapy. At delivery, 98.9% of newborns receive a HBV vaccine birth dose, and 82% received HBIG. At 12 months of age, 94.7% have received the scheduled HBV vaccines. Eight percent of infants born from followed-up women were HBsAg positive. The mother's HBeAg-positive status was associated with a higher risk of HBV infection in infants.

**Conclusion:**

The HBV prevalence and MTCT rates are high in Haiphong. A strong national plan to increase the access to preventive measures and to monitor results is needed in order to decrease this prevalence.

## 1. Introduction

An estimated 257 million people are living with hepatitis B virus (HBV) infection worldwide, 45% of them in the Western Pacific Region [1, 2]. Mother-to-child transmission (MTCT) accounts for the majority of new chronic HBV carriers, especially in Asia. HBV can be transmitted in utero, during delivery or during and after infancy [3–5]. About 80–90% of infants infected at birth will develop chronic HBV infection, and 20–30% of adults who are HBV chronically infected will experience serious complications including liver fibrosis, cirrhosis, hepatocellular carcinoma, and liver-related death [6]. In 2016, the World Health Assembly adopted the first global health targets for elimination of viral hepatitis as a public health threat. The objectives are a reduction of 90% of new infections and 65% of mortality by 2030, with an aim to reduce the prevalence of hepatitis B surface antigen (HBsAg) in children to 1% by 2020 and <0.1% by 2030 [7]. Although the growing political momentum for viral hepatitis was strongly welcomed by the hepatitis community and public health organizations, the targets were recognized as ambitious in scale. To prevent MTCT, the World Health Organization (WHO) recommends a universal immunization with at least 3 doses of HBV vaccines as a first-line prevention against perinatal infection for all infants. The first dose of vaccine is to be administered at birth, along with hyperimmune hepatitis B immunoglobulin (HBIG) to babies. More recently, it has been recommended to add antiviral therapy for HBV-infected pregnant mothers at high risk of infants' immunoprophylaxis failure despite receiving a HBV birth dose vaccination (BDV) and HBIG [8].

Vietnam accounts for approximately 9.6 million people living with chronic HBV infection [2, 9]. Chronic HBV is an important contributing factor to the development of primary liver cancer and cirrhosis, which is the most common cause of death in Vietnam [9, 10]. The reported prevalence of HBsAg in the general population ranged from 15 to 20% [11–13]. Current estimates suggest that 10.8% of the population in Vietnam are living with chronic hepatitis B [2]. HBV prevalence estimates in pregnant women consulting at an antenatal care facility range between 10% and 13% [13, 14]. Previous studies in different regions in Vietnam estimate the incidence of new HBV cases among the general population to be 10%-20% of the population each year, mainly through MTCT [11, 12, 15].

To prevent MTCT, Vietnam has introduced a 4-dose HBV vaccination schedule (at birth and at 2, 3, and 4 months after birth, free of charge) beginning in 2004 [11, 13]. In 2014, the Ministry of Health of Vietnam disseminated the “Guidelines for hepatitis B diagnostic treatment and prevention” in which a HBIG dose is recommended at birth for all children born from HBsAg-positive mothers and prophylactic HBV MTCT antiviral treatment was prescribed to women when HBV DNA ≥ 200,000 IU/mL (10^6^ copies/mL) [16, 17]. The antiviral treatment's cost is covered by health insurance, but the HBIG cost of nearly 100 USD/dose must be fully paid by the parents.

This study is aimed at assessing the prevalence of HBV infection among pregnant women consulting at the antenatal care clinic of the Gyneco-Obstetric Hospital of Haiphong city, the 3rd largest city of Vietnam, and evaluating the real-life use of prevention measures and the actual rates of MTCT.

## 2. Materials and Methods

### 2.1. Study Subject Sampling and Follow-Up

Haiphong Gyneco-Obstetric Hospital provides antenatal care and delivery services to about 4500 pregnant women annually from different urban and suburban districts of Haiphong city. Antenatal care consists of at least three antenatal consultations at the first, second, and third trimesters of gestation before delivery. The study design planned to screen approximately 1500 women at an early stage of pregnancy in order to detect about 150 HBV surface antigen- (HBsAg-) positive pregnant women, to follow them up, investigate their actual use of HBV preventive measures, and monitor the MTCT rate.

We invited all pregnant women attending Haiphong Gyneco-Obstetric Hospital from October 2017 to March 2018 for their early (<24 weeks) gestation consultation to participate in our study. The women who consented to participate were asked to be given a screening test for HBV infection, e.g., a blood HBsAg test. A short questionnaire was used to collect sociodemographic information such as age, education level, employment, average income, number of pregnancies, history of HBV vaccination, and relevant contact information. Pregnant women were then given an appointment for the next antenatal consultation at the hospital by the end of their second trimester of gestation.

At the next antenatal consultation, HBsAg-positive women were asked to consent to be followed up until 12 months after delivery. Women were interviewed about personal and family risk factors related to HBV infection such as history of HBV infection, husband/partner's HBV infection status, and information on any of their family members being infected with HBV. A blood sample was taken for measuring alanine aminotransferase (ALT) serum levels, HBV markers, and HBV deoxyribonucleic acid (DNA) load. The antiviral therapy indications for pregnant women were complied with the Vietnamese national “Guidelines for hepatitis B diagnostic treatment and prevention.” Specifically, Tenofovir (300 mg/day orally) was prescribed to HBsAg-positive women with an ALT serum level ≥two times the upper limit of the normal value (ULN) when HBV DNA > 20,000 IU/mL (10^5^ copies/mL) and if they tested positive for HBeAg or when HBV DNA > 2,000 IU/mL (10^4^ copies/mL) and they tested negative for HBeAg. Prophylactic HBV MTCT antiviral treatment was prescribed when HBV DNA ≥ 200,000 IU/mL (10^6^ copies/mL) [16, 17].

During their stay at the hospital for delivery, women were interviewed about their previous use of antiviral treatments. Information about the mode of delivery, anti-HBV vaccine dose given at birth, and HBV immunoglobulin immunization to the newborn was collected from medical records. Blood samples from mothers and newborns (umbilical cord) were taken to test HBV markers and HBV-DNA load.

At 6th and 12th month postpartum, women were contacted to schedule an appointment for a home visit. Information regarding the compliance with the infant's vaccination schedule and the mode of feeding the child since the last visit was collected through questionnaire-based interviews with the mother. A blood sample was taken from the infant at each visit to test for HBV markers.

### 2.2. Laboratory

Laboratory testing for HBsAg, HBe antigen (HBeAg), and HBV DNA load was performed at the Biomolecular Laboratory of Haiphong University of Medicine and Pharmacy. Measuring HBsAg and HBeAg was performed using an enzyme-linked immunosorbent assay (ELISA) technique on a PW 40, IPS, PR 2100 machine with reagents from Diagnostic Automation/Cortez Diagnostics, Inc., USA. Quantification of HBV DNA load was measured using real-time PCR (real-time polymerase chain reaction) using FTD Hepatitis B DNA from Fast-Track Diagnostics, Luxembourg, with a detection limit < 50 IU/mL. Alanine aminotransferase (ALT) levels were measured using the HumaStar 600 automate (Human company, Germany). Due to limited research budget, laboratory testing for anti-HBs and ALT levels in the infants was not performed.

### 2.3. Data Collection and Analyses

Data were collected using print Clinical Report Forms, double-entered into an Excel file (MS Office 365), and then imported and analyzed using STATA v13.0 (Stata Corp, College Station, TX, USA).

Prevalence of HBV infection among pregnant women was calculated as number of pregnant women with a positive HBsAg result among the women who attended Haiphong Gyneco-Obstetric Hospital for a prenatal care consultation during the collection of data period. The rate of HBV MTCT was calculated as number of infants with a positive HBsAg result among the total number of infants seen and tested at 12 months of age. The 95% confidence intervals (CI) of the proportions were calculated using the Clopper-Pearson method. Comparisons between groups of maternal and infant characteristics were assessed using Fisher's exact test for categorical variables and the Wilcoxon-Mann-Whitney test for continuous variables. Comparison of proportions of the same maternal and infant characteristics between two visits was assessed using the McNemar test for paired data. Factors associated with infant HBV infection were determined using univariate logistic regressions. Factors associated with infant HBV infection variable at *p* ≤ 0.20 in univariate analysis were assessed in a multivariable logistic regression model. Model fitness was assessed by using the linktest and the Hosmer-Lemeshow test. All tests were two-sided, and *p* < 0.05 was considered statistically significant.

### 2.4. Ethical Consideration

This study was approved by the Institutional Review Boards of the Haiphong University of Medicine and Pharmacy, Vietnam (No. 105/HDDD).

## 3. Results

### 3.1. Study Participants at Enrollment and Follow-Up

From October 2017 to March 2018, among 1735 pregnant women coming to Haiphong Gyneco-Obstetric Hospital for their early gestation (<week 24) consultation, 1721 (99%) consented to participate in our study. Their mean age was 30 years (SD 5.3, range 18–42 years). One hundred and thirty-three (10.6%) were HBsAg positive. All 183 participants consented to be followed up, and all were seen at their next antenatal consultation and delivery visits. Twenty-four (13.1%) had a high HBV DNA load (≥200,000 IU/mL). All women received a MTCT prophylactic antiviral therapy. Nine women met the HBV disease treatment criteria, but only 6 (66.7%) were treated. At delivery, all 183 followed-up women and their newborns were tested for HBV markers and HBV DNA load. At 6th and 12th month postpartum follow-up visits, 176 and 150 mother-infant pairs, respectively, received a home visit. Mothers were interviewed, and infant blood was taken for HBV testing. A total of 33 women-infant pairs (18.0%) who were lost during the 12-month postpartum follow-up (7 loss to follow-up during first 6 months and 26 loss to follow-up during the last 6 months) were those who could not be reached by phone neither or at their address. The baseline characteristics of 33 mothers lost to follow-up by 12-month postpartum were not significantly different from those who completed the study follow-up.

### 3.2. HBsAg Prevalence among Pregnant Women

Table 1 provides HBsAg prevalence among the 1721 pregnant women who participated in our study, according to their social-demographic characteristics. A total of 183 women tested HBsAg positive giving a prevalence rate of 10.6% (95% CI: 9.2-12.1). Among the 183 HBsAg-positive women, more than half (52.4%) had a college or a higher level of education. Most of them (82.5%) had an average income of 250 to 500 USD/month. More than half (56.3%) reported having been vaccinated against HBV, and more than a quarter (26.2%) knew that they were HBV infected before pregnancy. These proportions are equally distributed in all 1721 pregnant women participating in the study. In general, the HBsAg prevalence did not differ between different sociodemographic groups (*p* > 0.05).

### 3.3. HBeAg Status and Actual Use of Preventive Measures

A total of 42 women (23.0%) had a positive HBeAg test.  Table 2 shows some important features of infected women and their infants followed up up to 12 months of age, stratified by maternal HBeAg status. HBeAg-negative and HBeAg-positive women were about the same age (median age of 29 versus 30 years, *p* = 0.59). However, HBeAg-positive women had a higher ALT level (median of 91 U/L versus 29 U/L, *p* < 0.001). Forty-eight (26.2%) of 183 women had a detectable HBV DNA load, and 24 of them (13.1%) had a HBV DNA load higher than 200,000 IU/mL. The proportions were higher among HBeAg-positive women (80.1% and 52.4%) compared to HBeAg-negative women (9.9% and 1.4%, respectively), *p* < 0.001. Thirty-one women (16.9%) had been treated with antiviral drugs during their pregnancy representing 2.1% in the HBeAg-negative group and 66.7% in the HBeAg-positive group (*p* < 0.001). At delivery, 37 women (20.2%) still had a detectable HBV DNA load, with 6 (3.3%) having a very high HBV DNA level (≥200,000 IU/mL). The proportions did not differ between HBeAg-negative and HBeAg positive-groups (*p* > 0.05). A higher proportion of female infants were born to HBeAg-positive mothers compared to HBeAg-negative mothers (61.9% vs. 44.0%, *p* = 0.04). A high rate of infants (98.9%) received a BDV within 24 hours, and 82.0% received HBIG immunization at birth. At 12 months of age, 94.7% of infants had completed the scheduled HBV vaccine doses (Table 2).

### 3.4. Mother-to-Child Transmission

At 6-month postpartum, 176/183 infants were reachable. All had an undetectable HBV DNA load, and 23 infants (13.1%) were HBsAg positive. At 12 months of age, all 150 infants were seen. All had an undetectable HBV DNA load, and 12 infants (8.0%) were HBsAg positive. The prevalence of positive HBsAg was higher among infants born to HBeAg-positive mothers compared to those born to HBeAg-negative mothers (17.1% vs. 5.2%, *p* = 0.02).

Table 3 displays the factors associated with infant's HBsAg-positive status at the age of 12 months. The results show that the risk of being HBsAg positive increased dramatically in infants born to HBeAg-positive mothers (OR = 65.8; *p* < 0.001). Having any family member infected with HBV and not having completed HBV vaccination schedule were also associated with being HBsAg positive in univariate analyses although this did not become statistically significant in a multivariable analysis (*p* > 0.05).

Table 4 shows that among 12 infected children, 10 were born to HBeAg-positive mothers, 4 were born to mothers with a detectable HBV DNA load, 5 had at least one family member known to be infected, and 5 did not receive HBIG at birth.

One of the 22 infants born among the 24 women with a high HBV DNA level (>200.000 IU/mL) at the second trimester of gestation and who had been treated with antiviral drugs to prevent MTCT was HBsAg positive at 12 months of age (Table 4, case number 1). The mother of the affected child had a very high level of HBV DNA load (1,174,000 IU/mL) during her pregnancy. Her HBV DNA level had remained relatively high at delivery (82,400 IU/mL). Among 9 women who met the HBV antiviral treatment criteria for their HBV disease (HBV DNA load > 20,000 IU/mL, HBeAg positive and ALT serum level ≥ 2x ULN), 3 did not take the treatment, and 2 gave birth to HBsAg-positive infants (Table 4, case numbers 2 and 3). At delivery, one of these 2 mothers had a rebounded HBV DNA load (to 1,756,000 IU/mL) while one had similar HBV DNA levels (24,600 IU/mL) as before treatment.

## 4. Discussion

This observational cohort study carried out on pregnant women who were followed up at the Gyneco-Obstetric Hospital of Haiphong is aimed at evaluating the prevalence HBV infection among pregnant women, describing their use of preventive measures and the rate of MTCT in the real-life practice of the Vietnamese health care system.

The first observation is that the overall prevalence rate of HBsAg is 10.6% (95% CI: 9.2-12.1) among this sample of pregnant women. To our knowledge, our study is one of very few studies that report an updated prevalence of HBV infection among pregnant women in Vietnam. Most of the prevalence rates reported come from studies carried in the 1990s [13, 14]. Acknowledging that our study is not a national nor a regional prevalence survey, our sampling method may not represent a perfect population representativeness and may suffer from possible important selection bias. For instance, pregnant women in the sample tend to come from the city of Haiphong, one of the largest urban agglomerations of Vietnam. Women had a higher monthly income compared to the national level of <2800 USD/capita/year in 2019 and had a higher education level compared to those observed in other population studies [18–20]. Several studies have shown that a low socioeconomic status was a risk factor for HBV infection [21]. We can therefore assume that the situation of other provinces that are mostly rural and remote areas could have a higher HBsAg prevalence, as noted in some studies [11, 12, 18]. Most data collected in different locations in Vietnam showed prevalence rates of 15-20% in the general population [11–13] and 10-13% in pregnant women [13, 14]. Although our results are similar to recent estimates of HBV prevalence that suggest that about 10.8% of the population is HBsAg positive [2], these rates are much higher than the 8% threshold that defines a high endemicity level [22]. Interestingly, large differences are observed when comparing our results with those of recent surveys conducted among pregnant women in neighboring countries to Vietnam including China (5-6%) [23, 24], Cambodia (3-5%) [2, 25], and Laos (2.9%) [11], a country whose capital showed a significant decrease from 2008 to 2014 [26]. Our study results confirm that the current prevalence of HBV infection in Vietnam is still very high. HBV infection still remains a huge public health problem and will continue to be a future challenge unless the political and health system prioritizes the fight against this disease.

Twenty-three percent of HBsAg-positive women were HBeAg positive, and 13.1% had a HBV DNA load (>200,000 IU/mL) higher than the threshold above which the majority of MTCT is known to occur [8, 27]. The presence of HBeAg in HBsAg-positive pregnant women is an indicator of active viral replication; it increases the risk of perinatal transmission of HBV. The proportion of women with HBeAg (23%) among HBsAg-positive women was similar to that reported in recent studies in Asia [28–30]. In Southeast Asia, over 30% of HBsAg-positive women between 20 and 39 years are presumed to be HBeAg carriers [31].

In our study, 8% of infants born to infected mothers were HBsAg positive at 12 months of age, although all of them had an undetectable HBV DNA load. The rate is significantly higher among infants born to HBeAg-positive mothers compared to those born to HBeAg-negative mothers (17.1% vs. 5.2%, *p* = 0.02). In multivariable analyses, only the mother's HBeAg-positive status was statistically associated with the risk of the infant to be HBsAg positive (aOR = 65.8, *p* < 0.001). If we consider a positive HBsAg at 12 months of age as a proxy of an infant HBV infection, the infection rate among infants born to HBeAg-positive mothers in our study is higher than the 7% to 11% reported from most recent studies [32–35]. Studies in neighboring countries or regions reported lower rates of transmission: 2% in Thailand [36], 4% in Laos [28], and 4.5% in Hong Kong [37]. The differences observed above can be explained by the fact that most of the infection rates in these countries came from clinical trials while our study results reflect the rates of infection in a real-life practice.

**Figure 1:**
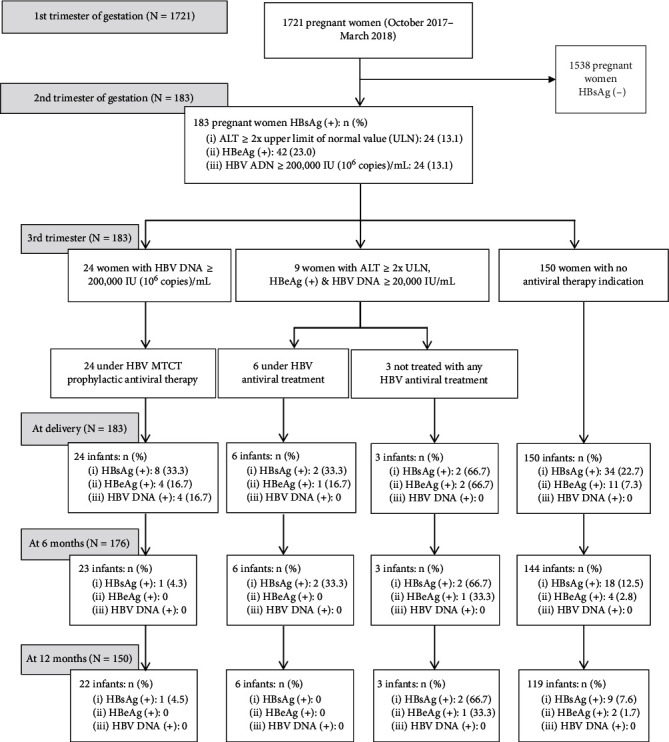
Participant enrollment and follow-up.

In our study, many HBeAg-positive mothers (e.g., mother-infant pairs 6 to 10 in Table 4) and HBeAg-positive infants (*n* = 5 at six months, and *n* = 3 at 12 months, Figure 1) had undetectable HBV DNA load. This finding is unusual, but actually, we could not have any reasonable explanation for this unusual pattern.

All 24 women with a high HBV DNA load (>200,000 IU/mL) had taken antiviral therapy to prevent MTCT. One of the 22 infants seen at 12 months of age (4.5%) was HBsAg positive. However, among the 9 women who needed to be treated for HBV disease, only 6 undertook antiviral treatment, and 3 were untreated. Two infants born from these 3 untreated women (66.7%) were HBsAg positive at 12 months of age. Moreover, 9 of 119 infants born to HBsAg-positive mothers who did not meet the indication criteria for treatment were HBsAg positive (7.6%) at 12 months of age despite the fact that most of the infants have received BDV (98.9%) and HBIG immunization (82%) and completed the HBV vaccination schedule (94.7%). Our results suggest that in order to achieve a successful reduction of MTCT, it may be relevant to review and evaluate the quality of the preventive measures instead of looking only at quantitative percentages. Data found in our study suggest that we are very far behind the WHO's targets to reduce the prevalence of HBsAg in children to 1% by 2020 and to <0.1% in 2030 [7]. We may need a national study on a representative population before considering reviewing the strategies and targets. In addition, a strong national plan to strengthen combined preventive measures with a good monitoring system is likely needed.

The rates of using preventive measures such as BDV, HBIG immunization, and HBV vaccination observed in Haiphong are higher than those observed in neighboring countries [28, 30, 38, 39] and around the world [8, 40, 41]. They are also higher compared to recent rates reported from other provinces in Vietnam [18–20] or from national estimates [40, 42, 43]. Different studies found that despite implementation of appropriate immunization programs, about 8–30% vertical transmission still occurs in infants born to HBeAg-positive women [27, 44, 45]. Infant scheduled vaccination interrupts horizontal (child-to-child) HBV transmission but does not effectively control HBV MTCT [8]. In China, despite the fact that 94% of children have received three doses of the hepatitis B vaccine, MTCT still accounts for 40–50% of new HBV infections [46].

Antiviral therapy given to mothers with a high HBV DNA load has been shown to be effective in preventing MTCT [8, 47–49]. Results from our study show that the infection rate among infants born from mothers who received antiviral treatment was lower compared to children whose mothers did not receive treatment. However, clinical assessment of the need of a prophylactic antiviral therapy is done at the second trimester of gestation or earlier. However, HBV load can change over time; HBV DNA load can be diagnosed as too low at the early trimester of gestation to justify a prophylactic treatment and can become high at the last trimester of the pregnancy and before delivery. These cases are therefore at high risk of MTCT. In our study, the proportion of women with high HBV DNA load (≥200,000 IU/mL) was reduced from 13% in the second trimester of gestation to 3% at delivery, but none of the women with a high HBV DNA load at delivery had a high HBV DNA load at the second trimester.

Scaling up interventions can benefit from experiences drawn from successful programs in countries with similar epidemiologic characteristics. China is an example of a high HBV burden country, which has made substantial progress in its efforts towards elimination of HBV MTCT. Sequential national serosurveys have shown a dramatic reduction in HBsAg prevalence among those 5-year-olds, from 9.7% in 1992 to 0.3% in 2014 [50]. This has been accomplished through strong political commitment with early introduction of universal BDV, HBIG immunization at birth, infant HBV vaccination, and peripartum antiviral therapy. Modelling studies suggest that continued coverage at such high levels could lead China to reach its <0.1% HBsAg prevalence target [8].

### 4.1. Limitations

One limitation was that our study was conducted in an urban area, and therefore, results may not be generalizable to the nationwide situation. Indeed, the sample consisted of women living in one of the largest urban areas of Vietnam, with easy access to a high-quality health care system and having the financial ability to pay for HBIG immunization. A second limitation was the low number of infected infants, limiting the possibility that the multivariable analyses would reveal more risk factors for transmission other than maternal HBeAg. On the other hand, the fact that anti-HBs and ALT levels were not performed has also prevented us from understanding further about the hepatitis and immune status of infants in their 12 months of age.

### 4.2. Implications

Our study results confirm that HBV infection remains a major public health problem in Vietnam. A strong national plan to strengthen combined preventive measures with a good monitoring system is needed. Firstly, improved access to HBV detection and to the identification of women at high risk of transmission to their infants (i.e., HBsAg and HBeAg positive who have a high HBV DNA level) can be implemented through an affordable point of care that provides rapid diagnostic tests (RDTs) for HBsAg and HBeAg. However, HBV DNA quantification is costly and accessible only in a few laboratories at central and provincial hospitals. Given the fact that the HBeAg is the main predictor for MTCT, a positive HBeAg test can act as a surrogate marker of HBV replication. Combining these 2 RDTs could be done in settings where HBV DNA quantification is not available, which has been shown in other studies [30, 38]. Furthermore, 7 out of 12 infected infants belonged to mothers who were HBeAg positive but did not meet the criteria of antiviral treatment, and among 42 HBeAg-positive women in our study, only 11 women did not fulfill the treatment criteria. The choice of treating all women with concurrent HBsAg- and HBeAg-positive tests may be a very good approach of MTCT prevention and should be considered in our current situation.

HBIG needs to be freely accessible to all infants whose mothers are HBsAg positive at delivery. Currently, an HBIG dose costs nearly 100 USD in Vietnam, and it must be fully paid by the parents. Thirty-three infants (18%) in our study did not take HBIG, mainly because of its cost. Forcing health insurance to cover this dose is needed.

Another important issue observed in this study is that most pregnant women did not receive enough counselling on preventive measures during their antenatal consultations. Specifically, 3 women who did not take antiviral treatment for their HBV disease said that they were afraid of harmful effects that a treatment taken during pregnancy could cause to their future babies. Only women with a very high HBV DNA level went on to take antiviral prophylactic therapy as they were told that treatment would prevent HBV transmission to their future babies. The problematic lack of knowledge, attitude, and practice regarding HBV prevention was confirmed in recent studies on both pregnant women and healthcare workers in Vietnam [20, 51]. Training programs for healthcare workers and educating pregnant women to increase knowledge and reduce misperception about HBV may be a key intervention to improve access to care and prevent MTCT and may help speed up reaching the objective of eliminating HBV.

## 5. Conclusion

In this first cohort study describing the prevalence of HBV infection in pregnant women and real-life HBV perinatal transmission in Vietnam, we observed a high prevalence of HBsAg among pregnant women and a high rate of MTCT in spite of antiviral and HBIG treatments. Data found in our study suggest that Vietnam is still far behind the WHO's targets to reduce the prevalence of HBsAg in children to 1% by 2020 and to <0.1% in 2030. A strong national plan to strengthen combined preventive measures with a good monitoring system is needed. Implementing immediate training programs for healthcare workers and educating pregnant women have the ability to improve access to care and prevention.

## Figures and Tables

**Table 1:** HBsAg prevalence in pregnant women according to their sociodemographic characteristics.

Respondent's demographics	Women tested (*N* = 1721), *n* (%)	HBsAg positive (*N* = 183), *n* (% [95% CI])	*p* value
Age groups (years)			0.994
Under 25 years	365 (21.2)	39 (10.7 [7.5-13.8])	
26-30 years	596 (34.6)	63 (10.6 [8.1-13.0])	
31-35 years	477 (27.7)	52 (10.9 [8.1-13.7])	
>35 years	283 (16.5)	29 (10.2 [6.7-13.8])	
Employment			0.969
Farming	66 (3.8)	6 (9.1 [2.1-16.1])	
Blue-collar worker	663 (38.5)	70 (10.6 [8.2-12.9])	
Clerk/admin/teacher	583 (33.9)	64 (10.9 [8.4-13.5])	
Small trade/housewife	409 (23.8)	43 (10.5 [7.5-13.5])	
Educational level			0.983
None-secondary	66 (3.8)	7 (10.6 [3.1-18.1])	
High school	763 (44.4)	80 (10.4 [8.3-12.7])	
College/university or higher	892 (51.8)	96 (10.8 [8.7-12.8])	
Number of pregnancies			0.982
1	650 (37.7)	68 (10.5 [8.1-12.8])	
2	836 (48.6)	90 (10.7 [8.7-12.9])	
≥3	235 (13.7)	25 (10.6 [6.7-14.6])	
Average income (USD/month/capita)			0.983
<250	102 (5.9)	11 (10.8 [4.7-16.8])	
250-<500	1428 (83.0)	151 (10.6 [8.9-12.2])	
≥500	191 (11.1)	21 (11.0 [6.5-15.4])	
Reporting being vaccinated against HBV			0.796
Yes	984 (57.2)	103 (10.5 [8.5-12.4])	
No	737 (42.8)	80 (10.8 [8.6-13.1])	
HBV infection known before pregnancy			0.903
Yes	445 (25.9)	48 (10.8 [7.9-13.7])	
No	1276 (74.1)	135 (10.6 [8.9-12.3])	

**Table 2:** Characteristics of HBV-infected women and their infants according to maternal HBeAg status^∗^.

Participant's characteristics	Total	Women HBeAg (-)	Women HBeAg (+)	*p* value
*Maternal characteristics at baseline*				
Number of women with data	183	141	42	
Age (years), median (IQR)	30 (26-33)	30 (26-33)	29 (26-34)	0.59
HBV infection known before pregnancy, *n* (%)	48 (26.2)	24 (17.0)	24 (57.1)	<0.001
Partner's HBV status, *n* (%)				
HBsAg positive	13 (7.1)	7 (5.0)	6 (14.3)	0.04
HBsAg negative	162 (88.5)	126 (89.3)	36 (85.7)	
Does not known	8 (4.4)	8 (5.7)	0	
Having family members infected with HBV, *n* (%)	23 (12.6)	17 (12.1)	6 (14.3)	0.7
ALT (U/L), median (IQR)	32 (27-40)	29 (25-36)	91 (34-108)	<0.001
HBV DNA level at 7th month of gestation, *n* (%)				
Detectable	48 (26.2)	14 (9.9)	34 (81.0)	<0.001
>200,000 IU/mL	24 (13.1)	2 (1.4)	22 (52.4)	<0.001
*Maternal characteristics at delivery*				
Number of women with data	183	141	42	
Under HBV antiviral therapy in last 3 months, *n* (%)	31 (16.9)	3 (9.7)	28 (90.3)	<0.001
Mode of delivery, *n* (%)				
Vaginal	120 (65.6)	95 (67.4)	25 (59.5)	0.35
Caesarean	63 (34.4)	46 (32.6)	17 (40.5)	
HBV DNA level at delivery, *n* (%)				
Detectable	37 (20.2)	24 (17.0)	13 (31.0)	0.35
≥200,000 IU/mL	6 (3.3)	5 (3.5)	1 (2.4)	0.99
*Infant characteristics at birth*				
Number infants with data	183	141	42	
Sex—female, *n* (%)	88 (48.1)	62 (44.0)	26 (61.9)	0.04
Weight (kilograms), median (IQR)	3.0 (2.8-3.2)	2.9 (2.8-3.2)	3.0 (2.8-3.3)	0.46
Child HBV birth dose vaccination, *n* (%)	181 (98.9)	139 (98.6)	42 (100.0)	0.99
Child HBIG immunization, *n* (%)	150 (82.0)	116 (82.3)	34 (81.0)	0.85
Positive HBsAg, *n* (%)	46 (25.1)	27 (19.1)	19 (45.2)	0.001
*Infant characteristics at 6 months of age*				
Number infants with data	176	135	41	
Completed HBV vaccination schedule, *n* (%)	71 (40.3)	51 (37.8)	20 (48.8)	0.21
Infant feeding during the last 6 months, *n* (%)				
Breast-fed only	90 (51.1)	75 (55.6)	15 (36.6)	0.08
Bottle-fed only	73 (41.5)	58 (38.5)	22 (51.2)	
Mixed (breast-fed and bottle-fed)	13 (7.4)	8 (5.9)	5 (12.2)	
Positive HBsAg, *n* (%)	23 (13.1)	13 (9.6)	10 (24.4)	0.01
*Infant characteristics at 12 months of age*				
Number infants with data	150	115	35	
Completed HBV vaccination schedule, *n* (%)	142 (94.7)	108 (93.9)	34 (97.1)	0.46
Infant feeding during the last 6 months, *n* (%)				
Breast-fed only	52 (34.7)	42 (36.5)	10 (28.6)	0.24
Bottle-fed (and/or other food)	47 (31.3)	32 (27.8)	15 (42.8)	
Mixed (breast-fed + bottle-fed and/or other food)	51 (34.0)	41 (35.7)	10 (28.6)	
Positive HBsAg, *n* (%)	12 (8.0)	6 (5.2)	6 (17.1)	0.02

^∗^HBeAg status was counted on women's blood test results at second trimester of gestation.

**Table 3:** Factors associated with infant's HBV infection at 12-month follow-up (*N* = 150).

Infant's characteristics		Infant with positive HBsAg, *n* (%)	Crude OR^a^, OR [95% CI]	Adjusted OR^b^, OR [95% CI]	*p* value^c^
Mother's HBeAg status at delivery	HBeAg (-)	2 (1.7)	Ref.	Ref.	<0.001
	HBeAg (+)	10 (34.5)	31.3 [6.4-154.1]	65.8 [7.3-594.1]	

Mother's HBV DNA level at delivery	<200,000 IU/mL (10^6^ copies/mL)	11 (7.6)	Ref.		
	≥200,000 IU/mL (10^6^ copies/mL)	1 (16.7)	2.4 [0.3-22.6]		

Mother's HBV peripartum antiviral therapy	Yes	1 (3.4)	Ref.		
	No	11 (9.1)	2.8 [0.3-22.6]		

Having any family members infected with HBV^d^	No	7 (5.5)	Ref.	Ref.	0.119
	Yes	5 (21.7)	4.8 [1.4-16.6]	4.0 [0.7-23.4]	

Mode of delivery	Vaginal	8 (8.0)	Ref.		
	Caesarean	4 (8.0)	1.0 [0.3-3.5]		

Infant's sex	Female	7 (9.7)	Ref.		
	Male	5 (6.4)	1.6 [0.5-5.2]		

Infant's HBV birth dose vaccination	Yes	11 (7.4)	Ref.	Ref.	0.057
	No	1 (50.0)	12.5 [0.7-212.9]	36.1 [0.9-1459.5]	

Infant's HBIG immunization	Yes	7 (5.7)	Ref.	Ref.	0.181
	No	5 (18.5)	3.8 [1.1-12.9]	3.4 [0.6-19.9]	

Infant's scheduled HBV vaccination	Completed	10 (7.0)	Ref.	Ref.	0.593
	Uncompleted	2 (25.0)	4.4 [0.8-24.7]	2.1 [0.1-31.2]	

Infant feeding (during 12 months of age)	Breast-fed only	4 (8.5)	Ref.		
	Bottle-fed (and/or other food)	3 (5.8)	0.7 [0.1-3.1]		
	Mixed (breast-fed+bottle-fed and/or other food)	5 (9.8)	1.2 [0.3-4.6]		

^a^Univariate analysis; ^b^multiple logistic regression; ^c^likelihood-ratio test; ^d^including infant's father HBV infection status. OR: odds ratio; CI: confidence interval. *α* < 0.05.

**Table 4:** Some characteristics of mother-infant pairs whose infants are HBV infected (*N* = 12).

No.	Mothers' characteristics							Infants' characteristics					
	Maternal HBeAg status at 7th month of gestation	HBV DNA level at 7th month of gestation^∗^	Antiviral treatment	Maternal HBeAg status at delivery	HBV DNA level at delivery^∗^	Family member infected with HBV^†^	Mode of delivery	Infant's sex	BDV^‡^	HBIG^#^	Completed HBV vaccine schedule	Infant feeding	Infant HBeAg status at 12 months of age
1	Positive	1,174,000	Yes	Positive	82,400	No	Caesarean	Female	Yes	Yes	Yes	Mixed	Negative
2	Positive	47,600	No	Positive	1,756,000	Yes	Caesarean	Female	Yes	Yes	Yes	Breast-fed	Positive
3	Positive	30,000	No	Positive	24,600	No	Vaginal	Female	Yes	No	Yes	Breast-fed	Negative
4	Positive	420	No	Positive	Undetectable	Yes	Caesarean	Male	Yes	Yes	No	Mixed	Negative
5	Negative	Undetectable	No	Positive	402	No	Vaginal	Female	Yes	No	Yes	Mixed	Negative
6	Positive	Undetectable	No	Positive	Undetectable	No	Vaginal	Male	Yes	Yes	Yes	Breast-fed	Negative
7	Negative	Undetectable	No	Positive	Undetectable	Yes	Vaginal	Female	Yes	No	No	Mixed	Negative
8	Positive	Undetectable	No	Positive	Undetectable	No	Vaginal	Male	Yes	No	Yes	Mixed	Positive
9	Negative	Undetectable	No	Positive	Undetectable	No	Vaginal	Male	Yes	Yes	Yes	Bottle-fed	Negative
10	Positive	Undetectable	No	Positive	Undetectable	No	Vaginal	Female	Yes	Yes	Yes	Breast-fed	Negative
11	Negative	Undetectable	No	Negative	Undetectable	Yes	Vaginal	Female	No	No	Yes	Bottle-fed	Negative
12	Negative	Undetectable	No	Negative	Undetectable	Yes	Caesarean	Male	Yes	Yes	Yes	Bottle-fed	Negative

^∗^Test results in IU/mL or undetectable (under limit of detection); ^†^having any family member known being HBV infected (including husband/partner); ^‡^BDV: birth dose vaccination; ^#^HBIG: hepatitis B immunoglobulin immunization.

## Data Availability

The EXCEL/STATA data used to support the findings of this study are available from the corresponding author upon request.

## Abbreviations

ALT: Alanine aminotransferase
BDV: Birth dose vaccination
DNA: Deoxyribonucleic acid
ELISA: Enzyme-linked immunosorbent assay
HBIG: Hepatitis B immunoglobulin
HBV: Hepatitis B virus
HBeAg: HBe antigen
HBsAg: HBV surface antigen
MTCT: Mother-to-child transmission
PCR: Polymerase chain reaction
ULN: Upper limit of the normal value.
